# Detection of mRNA Expression and Copy Number Variations Within the Goat *Fec*^*B*^ Gene Associated With Litter Size

**DOI:** 10.3389/fvets.2021.758705

**Published:** 2021-10-18

**Authors:** Yi Bi, Weijie Feng, Yuxin Kang, Ke Wang, Yuta Yang, Lei Qu, Hong Chen, Xianyong Lan, Chuanying Pan

**Affiliations:** ^1^Key Laboratory of Animal Genetics, Breeding and Reproduction of Shaanxi Provincial, College of Animal Science and Technology, Northwest A&F University, Xianyang, China; ^2^Shaanxi Provincial Engineering and Technology Research Center of Cashmere Goats, Yulin University, Yulin, China; ^3^Life Science Research Center, Yulin University, Yulin, China

**Keywords:** goat, *Fec*^*B*^ gene, mRNA expression, CNV, litter size

## Abstract

The Booroola fecundity (*Fec*^*B*^) gene, as the first major fecundity gene identified in Booroola sheep, has attracted careful attention. So far, previous research have uncovered the Fec^B^ mutation (Q249R) as the main mutation by virtue of which sheep exhibits multiple lambing phenomena. This mutation is now being intensively studied and widely used. However, such effect of the Fec^B^ mutation has not been applied to goats, and similar types of the *Fec*^*B*^ gene in goats still need to be studied. Thus, the current study attempted to verify potential mutations in the goat *Fec*^*B*^ gene as well as investigate their functions related to fecundity. First, *Fec*^*B*^ expression was investigated in six different goat tissues, and we found that *Fec*^*B*^ expression was highest in the mammary gland, followed by the ovary. Next, the influence of the *Fec*^*B*^ gene was analyzed from a new perspective, where five potential copy number variations (CNVs) (CNV1–5) within the *Fec*^*B*^ gene were identified for the first time, and then their effects on litter size were measured. Our results point out that CNV3 (*P* = 3.44E-4) and CNV5 (*P* = 0.034) could significantly influence the litter size of goats. Identically, the combination genotype of CNV3 and CNV5 which consisted of their dominant genotypes was also significantly associated with goat litter size (*P* = 7.80E-5). Hence, CNV3 and CNV5 could serve as potential DNA molecular markers applied to DNA editing and DNA microarray. Additionally, the abovementioned study has laid a theoretical foundation for the detection of potential fertility-related quantitative trait loci within the goat *Fec*^*B*^ gene.

## Introduction

The Booroola fecundity (*Fec*^*B*^) gene, also named *the bone morphogenetic protein receptor 1B (BMPR1B)* gene, was first recognized as a high-prolificacy major gene in Booroola sheep (1) due to the multi-lamb phenomenon contributed by the Fec^B^ mutation (Q249R) (2, 3). Moreover, as a receptor of bone morphogenetic proteins (BMPs) (4), it is involved in the regulation of follicle-stimulating hormone (FSH) levels, which regulates the ovulation rate in animals and humans (5, 6). In detail, the diminution of the BMP signal reduced by Q249R leads to an increase in follicle-stimulating hormone (FSHR) and luteinizing hormone receptor (LHR) density and also reduces apoptosis to increase ovulation (7–9). Furthermore, the attenuation of BMP signaling can maintain the reserve of primordial follicles while promoting follicular growth and ovulation, which contributes to the overall fertility of a female (10). Subsequently, a previous study showed that the Q249R mutation can regulate the expression of BMP/SMAD signaling and significantly upregulate the expression of fecundity-related genes such as STAR, BMP6, and BMP2 (11). Moreover, it can increase ovulation by suppressing the expression of SMAD6, which acts as an inhibitor of the BMP/SMAD signaling pathway (12).

To date, the *Fec*^*B*^ gene has been deeply studied in several breeds and strains of sheep such as Hu, Chinese Merino prolific meat strain, Suffolk, Dorset, and Charolais sheep breeds (13, 14), especially the Q249R locus which was successfully applied in sheep breeding as a significant DNA marker. Although only Q249R is regarded as the major mutation related to high fertility in sheep, there are also several identified mutations within the *Fec*^*B*^ gene worth studying—for instance, g.29362047T>C and g.29427689G>A were found to be significantly associated with litter size in Hu sheep (15). In addition to single-nucleotide polymorphisms (SNPs), five insertion/deletions (indels) (4, 10, 12, 17, and 23 bp) in the *BMPR1B* gene were also verified in Chinese Australian White sheep. However, among them, only the 10-bp indel significantly affected the sheep litter size (16). In goat, a previous study has revealed that Smad signaling, steroidogenesis, and cell viability in granulosa cells have been altered upon modulation of the *Fec*^*B*^ gene which was similar to that documented in sheep breeds carrying the Fec^B^ mutation (17). Moreover, several research were devoted to the detection of potential mutations in goat *Fec*^*B*^ gene, but no major high-fertility-related mutation was discovered in goat until now. In detail, C94T was identified to exert a significant influence on the litter size of Liaoning cashmere goats (18). Apart from that, three novel SNPs, including G773C, A775G, and G777A, have also been uncovered in Assam hill and Markhoz goats, respectively, but their specific functions still need to be studied (19, 20). Altogether, as a major fecundity-related gene, the *Fec*^*B*^ gene as well as its function has attracted careful attention (20–25), and a number of SNPs and indels within the *Fec*^*B*^ gene have also been examined to varying degrees (26–28). However, almost all studies have focused on sheep, while no major quantitative trait locus (QTL) has yet been identified in goats. Additionally, we found that all relevant research focus on SNPs and indels without paying attention to copy number variation (CNV), which is also one of the promising DNA markers.

Therefore, in this work, we aimed to investigate potential CNVs within the *BMPR1B* gene and, for the first time, measure their effects on goat litter size, thereby trying to provide information for DNA editing and microarray as well as laying the theoretical foundation for improving goat fecundity.

## Materials and Methods

### Animals, Total RNA, and Genomic DNA Isolation

In order to obtain RNA samples for further experiments, a total of six different tissues (corpus luteum, large follicle, skeletal muscle, uterus, ovary, and mammary gland) of the goat were collected (*n* = 3). Total RNA extraction was carried out using TRIzol total RNA extraction reagent (Takara, Dalian, China), following the instructions of the manufacturer. Then, 1% agarose gel electrophoresis in 6 × loading buffer was used to evaluate the integrity of total RNA. RNA was then quantified and qualitatively analyzed using a Nanodrop 2000 Spectrophotometer. Qualified RNA samples were stored at −80°C. To accomplish the further experiment, first-strand cDNA was synthesized by Prime Script™ RT Reagent Kit (Takara, Dalian, China). After that, these cDNAs were conserved at −20°C for subsequent experiments.

Shaanbei white cashmere (SBWC) female goats (2–3 years) that were healthy and under the same feeding and management conditions were used as the DNA sample in this work. All genomic DNAs were isolated from the ear tissue of 312 female goats according to the protocol of our previous study (29) and then stored at −40°C. In addition, all litter size and growth trait records of these goats were provided by the staff from farm records.

### RNA Experiment: mRNA Expression

A pair of primers for qPCR (FecB-F: F′-GTGTCAGGAGGTATAGTGGAAGAA-R′; FecB-R: F′- ACACACGATCTCTCTCATGCC-R′) was designed according to the mRNA sequence (accession number: NM_001285575.1) of the goat *Fec*^*B*^ gene in the NCBI database (https://www.ncbi.nlm.nih.gov/gene) *via* primer premier 6.0 (Table 1). It has covered all exons of the goat *Fec*^*B*^ gene to ensure cDNA amplification. The qPCR reaction system (10 μl) comprised of 5 μl of 2 × SYBR Premix Ex Taq (Takara Biotech, Dalian, China), 1 μl of cDNA, 0.5 μl of each primer, and 3 μl of ddH_2_O, using the same protocol as in our previous study (30). *GAPDH* was used as the reference gene (31). The results were then analyzed by 2^−Δ*ΔCt*^ method (32, 33).

**Table 1:** Information on the primers used in this study.

**Loci**	**Primer sequence (5^**′**^ to 3^**′**^)**	**Tm (^**°**^C)**	**Length**	**Location**
CNV1-F	TGAAAACAAGGAGGCAAGGAA	57.97	141 bp	Intron 1
CNV1-R	TAACCCTTCATCACCTTTCTCC	57.43		
CNV2-F	AGAGGCTGAGGTCTAAATTGTT	57.08	158 bp	Intron 1
CNV2-R	GACTGCTCATTTGTTGGTGGG	59.73		
CNV3-F	CAGATTTCAGCCTTTGCGGG	59.83	111 bp	Intron 1
CNV3-R	TTGGGGCAGTCAGGAAAGAG	59.60		
CNV4-F	CAGTCGTATCCTGGCACTGA	59.18	165 bp	Intron 1
CNV4-R	TGCCTTTAGGTCAGTGGGAAC	59.93		
CNV5-F	CCAAGGTAACCCAGAACTAGACACA	58.60	200 bp	Intron 2
CNV5-R	ACGACGACATCAGAGGGAGACA	58.90		
MC1R-F	GGCCTGAGAGGGGAATCACA	61.27	126 bp	–
MC1R-R	AGTGGGTCTCTGGATGGAGG	60.33		
Fec^B^-F	GTGTCAGGAGGTATAGTGGAAGAA	59.29	93 bp	–
Fec^B^-R	ACACACGATCTCTCTCATGCC	59.86		
GAPDH-F	AAAGTGGACATCGTCGCCAT	60.04	116 bp	–
GAPDH-R	CCGTTCTCTGCCTTGACTGT	59.97		

### DNA Experiment: Genotyping of CNV Within the *Fec^*B*^* Gene

**Figure 1:**
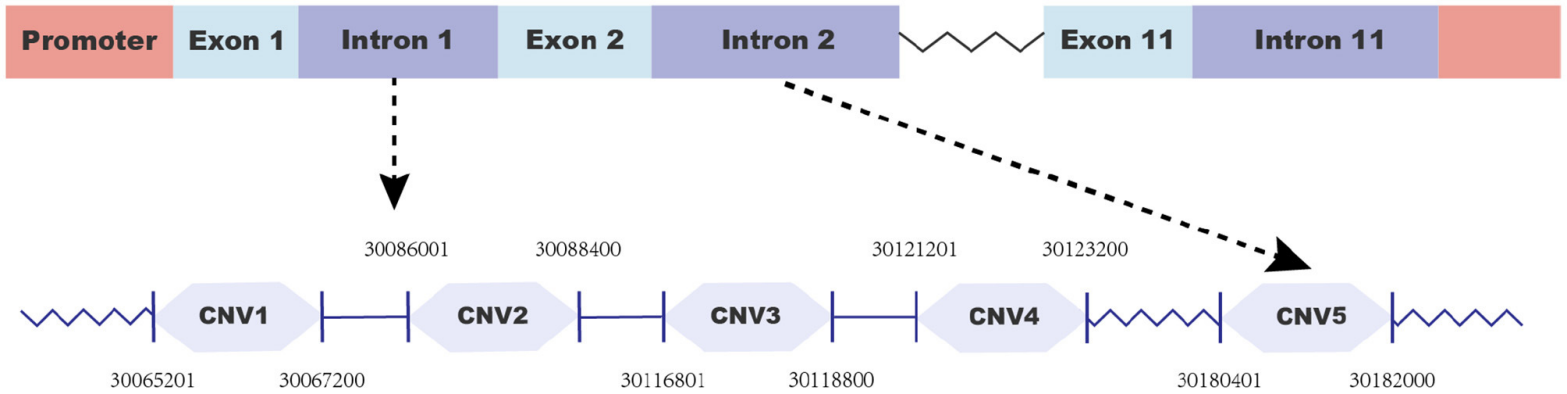
Gene structure of the goat *Fec*^*B*^ gene.

To identify copy number variations (CNVs), we first searched for potential CNVs in the goat *Fec*^*B*^ gene in the Animal Omics database (http://animal.nwsuaf.edu.cn) and found five CNV loci. Additionally, the figure of the goat *Fec*^*B*^ gene was drawn using Adobe Illustrator 2021 (Adobe, USA) as shown in Figure 1. When we analyzed these CNVs in our detected population, five pairs of primers (Table 1) were designed by NCBI (https://www.ncbi.nlm.nih.gov/tools/primer-blast), and qPCR was executed to further validate the CNVs. The qPCR reaction system (10 μl) contained 5 μl of 2 × SYBR Premix Ex Taq (Takara Biotech), 0.5 μl of goat genomic DNA, 0.5 μl of each primer, and 3.5 μl of ddH_2_O, using the program in our previous study (30). Then, the copy number of the goat *Fec*^*B*^ gene was calculated using 2^*^2^−Δ*Ct*^ method, where ΔCt = Ct target gene - Ct reference gene, and *MC1R* gene was the reference gene (28). Eventually, based on the value of 2^*^2^−Δ*Ct*^, the CNVs were divided into three types: “loss” (2^*^2^−Δ*Ct*^ <2), “median” (2^*^2^−Δ*Ct*^ = 2), and “gain” (2^*^2^−Δ*Ct*^ > 2).

### Statistical Analyses

The genotype frequencies of the five CNVs in the tested population was calculated, and the haplotypes were constructed *via* SHEsis online program (http://analysis.bio-x.cn). After that, the genotype distributions of these CNVs in all single lamb and multi-lamb individuals were analyzed using chi-square test (χ^2^).

Besides that, a linear model was used to estimate the effect of fixed factors on goat litter size (28): *Y*_ijk_ = μ + *a*_i_ + ß_j_ + *e*_ijk_, where *Y*_ijk_ is the litter size, μ is the overall mean value for each trait, *a*_i_ is the fixed-factor age, ß_j_ is the fixed-factor genotype, and *e*_ijk_ is the random error. For CNVs that displayed more than two genotypes, one-way ANOVA was used to assess their relationship with litter size; for <2 group, *t*-test was performed.

## Results

### mRNA Expression

**Figure 2:**
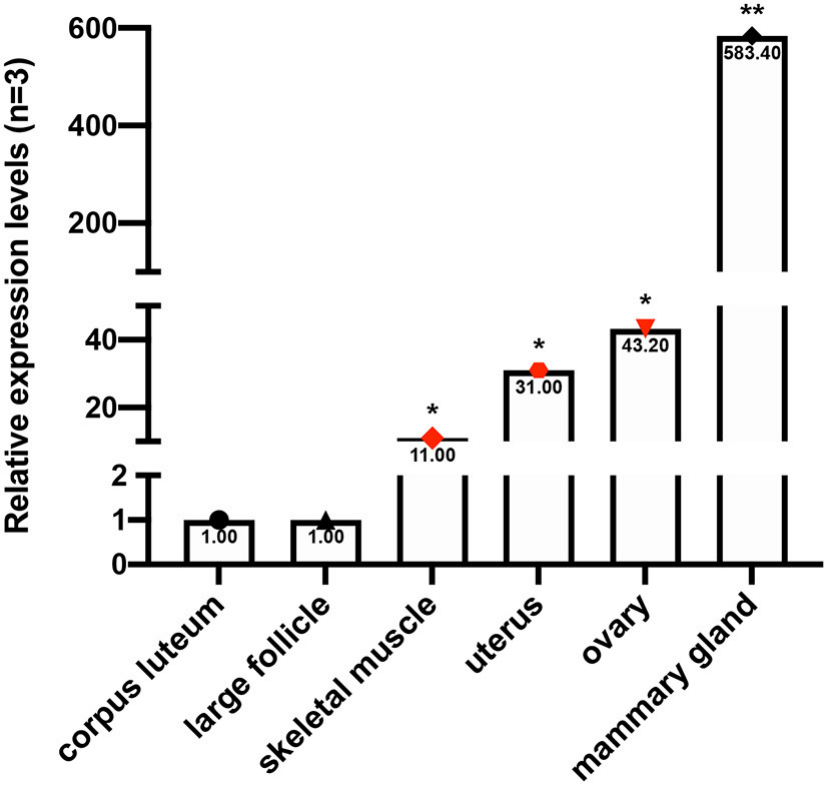
The mRNA expression profile of goat *Fec*^*B*^ gene, using the corpus luteum and large follicle as references (selecting three individuals for each tissue with three parallel repeats). *P < 0.05; **P < 0.01.

mRNA expression was determined in the mammary gland, corpus luteum, large follicle, skeletal muscle, ovary, and uterus tissues. Our results demonstrated that *Fec*^*B*^ expression was highest in the mammary gland and was 10-fold higher than that in other tissues. To add, the relative expression of the goat *Fec*^*B*^ gene in the skeletal muscle, uterus, and ovary varied from 10- to 50-fold changes compared to those in the corpus luteum and large follicle which were used as control (Figure 2).

### CNV Detection: Genotype Frequency, LD Analysis, and Haplotype Construction

To make the detected CNVs easier to describe, all of them were named according to their location at the goat chromosome as CNV1–5 (Table 2). Moreover, CNV2–5 displayed three genotypes (“loss,” “median,” and “gain”), while CNV1 only manifested two genotypes (“median” and “gain”) (Table 3). Furthermore, “gain” was the dominant genotype among all of them.

**Table 2:** Information on the copy number variations within the goat *Fec*^*B*^ gene.

**Loci**	**Chromas**	**Start**	**End**	**Length**
CNV1	6	30065201	30067200	2,000
CNV2	6	30086001	30088400	2,400
CNV3	6	30116801	30118800	2,000
CNV4	6	30121201	30123200	2,000
CNV5	6	30180401	30182000	1,600

**Table 3:** Typical frequencies of copy number variations within *Fec*^*B*^ gene in Shaanbei white cashmere goats.

**Loci**	**Sizes**	**Typic frequencies**		
		**Loss**	**Median**	**Gain**
CNV1	*n* = 112	0 (*n* = 0)	0.196 (*n* = 22)	0.804 (*n* = 90)
CNV2	*n* = 312	0.147 (*n* = 46)	0.128 (*n* = 40)	0.725 (*n* = 226)
CNV3	*n* = 313	0.153 (*n* = 48)	0.300 (*n* = 94)	0.547 (*n* = 171)
CNV4	*n* = 229	0.223 (*n* = 51)	0.332 (*n* = 76)	0.445 (*n* = 102)
CNV5	*n* = 302	0.013 (*n* = 4)	0.017 (*n* = 5)	0.970 (*n* = 283)

Hereafter, eight haplotypes were constructed among these five CNVs. In detail, G_1_G_2_G_3_G_4_G_5_, of which the frequency was 0.251, accounted for the most, while the frequencies of G_1_G_2_G_3_M_4_G_5_, G_1_G_2_M_3_G_4_G_5_, G_1_G_2_G_3_M_4_G_5_, M_1_G_2_G_3_M_4_G_5_, M_1_G_2_M_3_M_4_G_5_, M_1_G_2_L_3_L_4_G_5_, and M_1_M_2_L_3_M_4_G_5_ were 0.125, 0.188, 0.188, 0.125, 0.062, 0.062, 0.062, and 0.062, respectively (Table 4).

**Table 4:** Haplotype frequencies of the five copy number variations within the goat *Fec*^*B*^ gene.

**Haplotype**	**CNV1**	**CNV2**	**CNV3**	**CNV4**	**CNV5**	**Frequencies**
Hap 1	M_1_	M_2_	L_3_	M_4_	G_5_	0.062
Hap 2	M_1_	G_2_	L_3_	L_4_	G_5_	0.062
Hap 3	M_1_	G_2_	M_3_	M_4_	G_5_	0.062
Hap 4	M_1_	G_2_	G_3_	M_4_	G_5_	0.062
Hap 5	G_1_	G_2_	G_3_	M_4_	G_5_	0.125
Hap 6	G_1_	G_2_	M_3_	G_4_	G_5_	0.188
Hap 7	G_1_	G_2_	G_3_	M_4_	G_5_	0.188
Hap 8	G_1_	G_2_	G_3_	G_4_	G_5_	0.251

### Association Analysis

**Figure 3:**
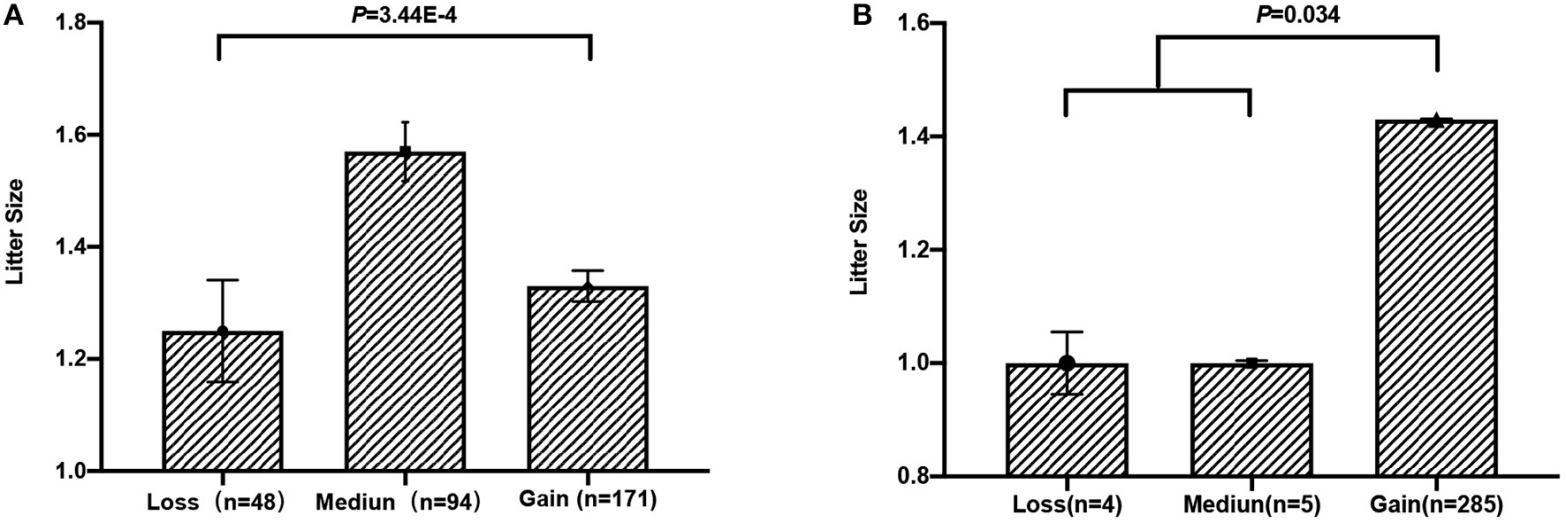
Association analysis between litter size and copy number variations (CNVs) in Shaanbei white cashmere goats. **(A)** Association between CNV3 and litter size. **(B)** Association between CNV5 and litter size.

**Figure 4:**
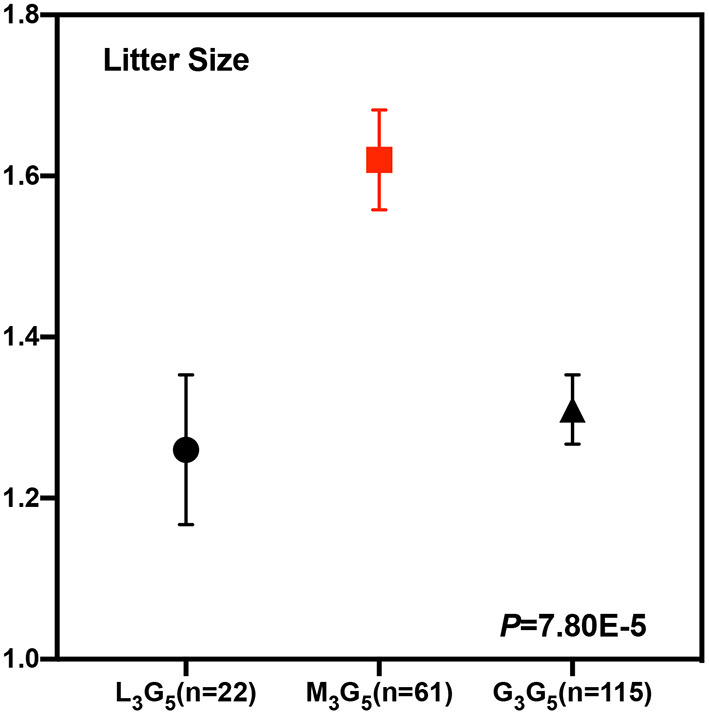
Least squares mean and standard error for litter size of the different combination genotypes of CNV3 and CNV5 within the *Fec*^*B*^ gene in Shaanbei white cashmere goats. Combination genotypes with a sample size of less than five were not calculated.

To test whether these five CNVs exerted a significant effect on the litter size of goats, we analyzed the association between them and the litter. Our results point out that CNV3 (*P* = 3.44E-4) and CNV5 (*P* = 0.034) were significantly associated with goat litter size (Figure 3; Table 5). The dominant genotype for CNV3 was “median,” while the dominant genotype for CNV5 was “gain.” Interestingly, the dominant combination genotype M_3_G_5_ could also have a significant influence on litter size (*P* = 7.80E-5) (Figure 4; Table 6). Additionally, the distribution of different genotypes in different litter-type populations significantly differed from each other (Table 7).

**Table 5:** Association analyses between litter size and copy number variation types in Shaanbei white cashmere goats.

**Locus**	**Genotype (LSM** **±** **SE)**			***P*-values**
	**Loss**	**Median**	**Gain**	
CNV3	1.25 ± 0.63^B^ (*n* = 48)	1.57 ± 0.51^A^ (*n* = 94)	1.33 ± 0.36^B^ (*n* = 171)	3.44E-4
CNV5	1.00 ± 0.11^b^ (*n* = 4)	1.00 ± 0.01^b^ (*n* = 5)	1.43 ± 0.02^a^ (*n* = 284)	0.034

*Values with different letters (A, B/a, b) within the same row differ significantly at P < 0.01/P < 0.05*.

**Table 6:** Least squares mean and standard error for litter size of different combination genotypes of CNV3 and CNV5 within the *Fec*^*B*^ gene in Shaanbei white cashmere goats.

**Genotype (LSM** **±** **SE)**			***P*-value**
**L_**3**_G_**5**_**	**M_**3**_G_**5**_**	**G_**3**_G_**5**_**	
1.00 ± 0.01^B^ (*n* = 22)	1.36 ± 0.13^A^ (*n* = 61)	1.67 ± 0.06^A^ (*n* = 115)	3.33E-14

*Values with different letters (A, B) within the same row differ significantly at P < 0.01. Combination genotypes with a sample size of less than five were not calculated*.

**Table 7:** Type distribution between mothers of single lamb and multi-lamb in Shaanbei white cashmere goats.

**CNV loci**	**Genotypes**	**Single lamb**	**Multi-lamb**	**Independent χ^**2**^*P*-values**
CNV1	Loss	1	0	χ^2^ = 3.372df = 2, *P* = 0.155
	Median	13	9	
	Gain	36	53	
CNV2	Loss	24	22	χ^2^ = 2.237df = 2, *P* = 0.327
	Median	27	13	
	Gain	139	87	
CNV3	Loss	36	12	χ^2^ = 20.213df = 2, *P* = 4.1E-5
	Median	40	54	
	Gain	115	56	
CNV4	Loss	28	29	χ^2^ = 0.436df = 2, *P* = 0.804
	Median	35	29	
	Gain	58	50	
CNV5	Loss	4	0	χ^2^ = 6.759df = 2, *P* = 0.034
	Median	5	0	
	Gain	160	123	

## Discussion

The *Fec*^*B*^ gene, which serves as a major gene for high fecundity, has been shown to contribute to the multi-lambing phenomena in sheep. Currently, it is gradually becoming clear how the *Fec*^*B*^ gene regulates sheep fecundity, while the specific mechanism of the goat *Fec*^*B*^ gene is still poorly understood and the first major QTL still needs to be disclosed. Here we identified the expression of the *Fec*^*B*^ gene in six different goat tissues and strikingly found that it had the highest expression in the mammary gland, indicating that the *Fec*^*B*^ gene might also function as a lactation regulator of goats. According to a previous study, an increase in litter size stimulates the expression of RFamide-related peptide mRNA, which might contribute to lactational anestrus in rats (34). Additionally, as the litter size increases, milk yield increases (35–38), and so does the uptake of net daily amino acid by the mammary gland, which is the basis for milk yield and lactoprotein production (39). In short, the above results indicate that the lactation would be mediated by an increase in litter size. Thus, the *Fec*^*B*^ gene, which significantly affects litter size, also presumably has a function related to lactation in goats. This might be the reason why the *Fec*^*B*^ gene had the highest expression in the mammary gland in goats. To our best knowledge, this is the first time that the *Fec*^*B*^ gene has shown such a high expression in the mammary gland, which leads to an indication that it is worth investigating whether the *Fec*^*B*^ gene has a lactation-related function, especially in populations with multiple lambs. Then, following the mammary gland, high expression in the ovary indicated that the *Fec*^*B*^ gene might be involved in ovarian activity, thereby influencing goat fertility. However, whether the *Fec*^*B*^ gene could impact goat fecundity has not yet been sufficiently studied (17).

Until now, reported studies have found a series of polymorphisms associated with fecundity within the *Fec*^*B*^ gene in sheep. However, related research analyzing the association between mutations in the *Fec*^*B*^ gene and fertility in goat are relatively scanty—for instance, G773C was identified to be unique in Assam hill goat, while the association between goat fecundity still needed to be studied (19). T242C was uncovered in Barbari, Beetal, Black Bengal, Ganjam, Jhakana, Osmanabadi as well as Sangamneri goat breeds, but the effect of genotypes was non-significant on litter size (40). Moreover, C94T within the goat *Fec*^*B*^ gene was revealed to be significantly associated with litter size in Liaoning cashmere goats (18). Interestingly, a related work investigated the Fec^B^ mutation in goat since its significant influence on multiple lambing in sheep. However, the results pointed out that the FecB mutation did not exert the same effect in goat, which led to a hint that novel significant mutations still need discovering (20). Many reports altogether revealed lots of polymorphisms within the *Fec*^*B*^ gene associated with fecundity in sheep, while potential mutations still need discovery in goats. No related research also paid attention on the CNV within this gene. In this study, we first identified five potential CNVs in the *Fec*^*B*^ gene and measured their effects on litter size in goats. Our results pointed out that the “gain” genotype, rather than another two genotypes, displayed the highest frequency in all five CNVs. Identically, the G_1_G_2_G_3_G_4_G_5_ haplotype was also the dominant haplotype with the highest frequency. In addition to the distribution of the five CNVs, we further analyzed their effect on goat litter size, which was the main purpose of this work. The results based on a large experimental population showed that CNV3 and CNV5 exerted significant effects on litter size in goats (*P* < 0.05), with “median” and “Gain” displaying a superior phenotype, respectively. In addition, the combination genotypes of CNV3 and CNV5 could also have the same effects on goat litter size. Notably, the combined genotype of “median” (CNV3) and “gain” (CNV5) performed superior litter size, which is consistent with the abovementioned results. Thus, CNV3 and CNV5 could serve as effective DNA markers applied to marker-assisted selection breeding, and individuals, especially with combined genotype “M_3_G_5_” should be selected to improve the litter size of goats.

Given that previous investigations revealing intronic variations could influence the interaction between transcription factors and host genes, we hypothesized that CNV3 and CNV5 might influence the combination ability of DNA sequence with transcription factors to indirectly influence the expression of the *Fec*^*B*^ gene (41, 42). To add, with BMPs acting as the key intraovarian factors regulating ovarian function (43–45), their biological effects will be mediated after binding to membrane-bound receptors. Mutations might influence the process BMPs combing with DNA sequence, thereby changing the related functions. However, how the two CNVs within the *Fec*^*B*^ gene influence the goat litter size still needs to be studied.

In conclusion, we have identified five CNVs within the *Fec*^*B*^ gene in SBWC goat population and found that CNV3 and CNV5 significantly influenced the litter size of goats. Moreover, this is the first report on the effect of CNVs within the *Fec*^*B*^ gene on litter size. Despite this, the specific mechanisms require further investigation.

## Data Availability Statement

The original contributions presented in the study are included in the article/supplementary material, further inquiries can be directed to the corresponding author/s.

## Ethics Statement

The animal study was reviewed and approved by the International Animal Care and Use Committee of the Northwest A&F University (IACUC-NWAFU; protocol number NWAFAC1008).

## Author Contributions

YB, XL, and CP came up with idea and revised the manuscript. YB wrote the manuscript and performed the experiments. WF, YK, KW, and YY collected the goat samples and isolated the genomic DNA. YB, YK, and LQ analyzed the data. All authors approved the final version of the manuscript for submission and contributed to the article and approved the submitted version.

## Funding

This work was supported by the National Scientific and Technological Innovation Project of Undergraduate of Northwest A&F University (No. 202106001) and the National Science Foundation of China (No. 32060734).

## Conflict of Interest

The authors declare that the research was conducted in the absence of any commercial or financial relationships that could be construed as a potential conflict of interest.

## Publisher's Note

All claims expressed in this article are solely those of the authors and do not necessarily represent those of their affiliated organizations, or those of the publisher, the editors and the reviewers. Any product that may be evaluated in this article, or claim that may be made by its manufacturer, is not guaranteed or endorsed by the publisher.
